# Clinical significance of long non‐coding RNA ZEB2‐AS1 and EMT‐related markers in ductal and lobular breast cancer

**DOI:** 10.1002/cnr2.1826

**Published:** 2023-04-23

**Authors:** Mahboobeh Zarei, Kianoosh Malekzadeh, Mahmoud Omidi, Pegah Mousavi

**Affiliations:** ^1^ Department of Medical Genetics, Faculty of Medicine Hormozgan University of Medical Sciences Bandar Abbas Iran; ^2^ Department of Pharmacology & Toxicology, Faculty of Pharmacy Hormozgan University of Medical Sciences Bandar Abbas Iran; ^3^ Molecular Medicine Research Center, Hormozgan Health Institute Hormozgan University of Medical Sciences Bandar Abbas Iran

**Keywords:** biomarker, breast cancer, EMT, LncRNA ZEB2‐AS1, non‐coding RNA

## Abstract

**Background:**

Breast cancer is considered the most prevalent type of cancer in women and accounts for a high rate of death. A body of research has demonstrated that lncRNAs have a regulatory function in human diseases, especially cancers. ZEB2‐AS1 is known as an oncogenic lncRNA in various types of cancers, and its deregulation may contribute to cancer development and progression. Therefore, we aimed to reveal the association of ZEB2‐AS1 expression with epithelial–mesenchymal transition (EMT) markers, as a hallmark of cancer progression, in a clinical setting.

**Methods:**

A recent study suggested that ZEB2‐AS1 is significantly involved in EMT. Here we intended to explore the roles of lncRNA ZEB2‐AS1 in breast cancer (BC) using bioinformatics tools and laboratory settings. We first evaluated the expression of ZEB2‐AS1 mRNA in tumor and healthy control tissues by lnCAR database. Furthermore, ZEB2‐AS1 expression level, ZEB2, E‐cadherin, and vimentin was measured via qRT‐PCR in 30 paired ductal and lobular carcinoma tissues from breast cancer patients and the normal adjacent ones. The correlation between the lncRNA ZEB2‐AS1 expression and clinicopathological characteristics of the breast cancer patients was evaluated.

**Results:**

ZEB2‐AS1 showed an upregulation in breast cancer tissues (*p* = .04) compared to normal adjacent samples. In addition, its level was higher in breast cancer patients with advanced Stages (III & IV) (*n* = 18) compared to early Stages (I & II) (*n* = 12) (*p* = .04). Moreover, ZEB2 (*p* = .01) and vimentin (*p* = .02) expression were upregulated in the BC sample, but the expression level of E‐cadherin (*p* = .02) was downregulated when compared with the adjacent normal tissues. By comparison of the expression of EMT‐markers between different stages of breast cancer, overexpression of ZEB2 (*p* = .04) and vimentin (*p* = .04) and down expression of E‐cadherin (*p* = .03) was observed in advance stages.

**Conclusions:**

Collectively, our findings suggest that ZEB2‐AS1 expression is significantly upregulated in tumor tissues, especially in advanced stages and ZEB2‐AS1 is associated with the aggressiveness of tumors by functioning as putative oncogenic lncRNA. In addition, a combination of ZEB2‐AS1 and these EMT markers in breast cancer potentiates these genes as biomarkers for tumor progression.

## 1 INTRODUCTION

Breast cancer (BC) is among the highly prevalent causes of mortality in women. Despite advanced diagnostic and therapeutic methods, the prognostic attempts for this type of cancer remain disappointing.^1^ Breast cancer cells are invasive and often have distant metastasis.^2^ Metastasis is one of the causes of treatment failure. Hence, comprehension of the mechanism of cancer progression can be useful in managing breast cancer.^1^

The long non‐coding RNA (lncRNA) is a member of non‐protein‐coding RNAs, the length of which is more than 200 nt. LncRNAs are capable of regulating gene expression at the transcriptional and post‐transcriptional levels. LncRNAs have a functional role in malignancy development, mainly in migration and invasion.^3^, ^4^

Several lines of studies have evidenced the aberrant expression and functional deregulation of lncRNAs in various malignancies including pancreatic cancer and hepatocellular carcinoma (HCC). It has been reported that lncRNA dysfunction might be significantly contributing to the development of breast cancer.^5^ For example, LncRNA DANCR,^6^ and LncRNA NEAT1^7^ contribute to tumor progression; by elevating proliferation, migration, and epithelial–mesenchymal transition (EMT) in breast cancer.

EMT is considered a mechanism that causes the progression and migration of tumors. Epithelial cells obtain specifications of mesenchymal cells via EMT. In EMT, we observe deregulation in cell–cell junctional proteins such as E‐cadherin (downregulation), vimentin and N‐cadherin (upregulation).^8^

ZEB2‐AS1 is a natural antisense transcript of the 5′UTR of zinc finger e‐box binding homologous box 2 (ZEB2). Studies indicated that ZEB2‐AS1 promotes cancer progression in multiple tumor types including bladder cancer,^9^ gastric cancer,^10^ and triple‐negative breast cancer.^11^ ZEB2‐AS1 can bind to donor splice site of ZEB2 mRNA in the cytoplasm and facilitate translation of ZEB2. ZEB2 has an inhibitory role in the E‐cadherin gene and therefore may have an inductive effect on EMT.^12^

This study aimed to investigate ZEB2‐AS1, ZEB2, E‐cadherin, and vimentin expression in human breast cancer patients and expression changes in these genes at different stages of BC. In the current research, we first evaluated the transcriptional patterns of lncRNA ZEB2‐AS1 breast cancer employing online bioinformatics tools. Furthermore, the present study analyzed the diagnostic and prognostic capabilities of lncRNA ZEB2‐AS1 mRNA expression in BC patients. To validate the data, in an independent patient cohort, we compared the expression changes of ZEB2‐AS1, ZEB2, E‐cadherin, and vimentin in human BC samples from different stages of breast cancer.

## 2 MATERIALS AND METHODS

### 2.1 Expression profile analysis

The research obtained the ZEB2‐AS1 expression pattern in ductal breast cancer and adjacent normal tissue using datasets provided by the lnCAR database (https://lncar.renlab.org/).^13^ lnCAR is a universal database that precisely displays differential expression profiles and prognostic landscape in human cancers by re‐annotating microarray probes. This server provides expression analysis for 54 893 samples across 10 cancers based on microarray from the GEO database.

### 2.2 Overall survival analysis

The GEPIA2 (http://gepia2.cancer-pku.cn/#survival) accomplishes survival analyses using the expression levels of the gene or isoform.^14^ The prognostic effects of gene expression level can be checked by users and the large TCGA and GTEx datasets can be searched too. The GEPIA2 web tool was employed to test the correlation of ZEB2‐AS1, the overall survival (OS), and Relapse‐Free Survival (RFS) in BC patients. In addition, we applied the survival map segment that compares the survival contribution of ZEB2‐AS1, ZEB2, vimentin, and E‐cadherin in OS and RFS of BC patients, calculated via hazard ratio (HR) based on the Mantel‐Cox test based on their gene expression analysis and survival time (Month). We considered levels (75%) and (25%) as the expression threshold for classifying high and low expression categories, respectively. The marked box around the tiles indicates statistical significance (*p* < .05) in cancer types. Poor and good prognoses are indicated by red and blue colors, and the color intensity indicates the HR level.

### 2.3 In silico analysis of correlation

Besides, we assessed correlation analysis between ZEB2‐AS1 and EMT‐related markers ZEB2, Vimentin, and E‐cadherin using StarBase 3.0 online server. StarBase 3.0 (http://starbase.sysu.edu.cn/) is an interactive web resource for large‐scale analysis of the RNA–RNA and protein–RNA interplay networks and can be used for correlation analysis between two genes (mRNAs, lncRNAs, miRNAs, and pseudogenes) in multiple cancer types.^15^

### 2.4 Clinical specimens

We obtained 30 pairs of ductal and lobular carcinoma BC tissues (12 ER^+^, 9 PR^+^, and 9 HER2^+^) as well as adjacent normal tissues from the Emam Khomeini Hospital affiliated with the Tehran University of Medical Science. We used liquid nitrogen to freeze all the fresh specimens. All patients signed the written informed letter of consent. The patients had not received the treatment previously, and carcinoma was approved by pathological diagnostic tools by expert pathologists who resided in the hospital. This project was confirmed by the Ethics Committee of the Hormozgan University of Medical Science (#IR.HUMS.REC.1398.038) (Table 1).

**TABLE 1:** Clinical information of patient samples.

Parameter	Cases (*n*)	Control (*n*)
Age (50 ± 10.5)		
<50	16 (47.1%)	16 (47.1%)
≥50	18 (52.9%)	18 (52.9%)
Stage		
I, II	14 (41.9%)	‐
III, IV	17 (58.06%)	
Grade		
I, II	22 (70.9%)	‐
III, IV	9 (29.03%)	
Tumor size		
>5	9 (29.03%)	‐
<5	22 (70.9%)	
Lymph node metastasis		
Yes	17 (58.06%)	‐
No	14 (41.9%)	30 (100%)

Abbreviation: *n*, number.

### 2.5 RNA extraction and qRT‐PCR

We isolated the total RNA from tissue specimens utilizing the TRIzol reagent (GeneAll, Korea) according to the manufacturer's protocol. The RNA samples were then reversely transcribed to cDNA using a PrimeScript RT reagent kit (Takara, Japan). Next, we measured the gene expression via a quantitative real‐time polymerase chain reaction (qRT‐PCR) with the SYBR‐Green Master Mix (Ampliqon, Denmark). Each sample was performed in triplicate. We considered GAPDH as the endogenous control. Gene expression levels were normalized to GAPDH by using the 2^−ΔΔct^ method. Table 2 enlists the primer sequences.

**TABLE 2:** Primer sequence.

Gene	Primer	Primer sequence
ZEB2‐AS1	Forward primer	5′‐TGCAGAGCAGGAGAGAGAC‐3′
	Revers primer	5′‐CACACCCTAATACACATGCCCT‐3′
ZEB2	Forward primer	5′‐TTCCTGGGCTACGACCATACC‐3′
	Revers primer	5′‐CAAGCAATTCTCCCTGAAATCC‐3′
E‐cadherin	Forward primer	5′‐TACAATGCCGCCATCGCTTA‐3′
	Revers primer	5′‐CGTAGGGAAACTCTCTCGGT‐3′
Vimentin	Forward primer	5′‐ATCCAAGTTTGCTGACCTCTCTGA‐3′
	Revers primer	5′‐ACTGCACCTGTCTCCGGTACTC‐3′
GAPDH	Forward primer	5′‐ATCAGCAATGCCTCCTGCAC‐3′
	Revers primer	5′‐TGGTCATGAGTCCTTCCACG‐3′

### 2.6 Statistical analysis

For data analysis, GraphPad Prism 8 was used. The difference between groups was analyzed using Student's *t*‐test (differences between 2 groups) and ANOVA (among several groups). A *p‐*value under .05 was interpreted as significant in all data. For depicted receiver operating characteristic (ROC) curve, we used SPSS software (IBM SPSS statistics 26). The combination ROC was obtained using regression logistics and enter model.

## 3 RESULTS

### 3.1 ZEB2‐AS1 expression level in BC tissues

**FIGURE 1:**
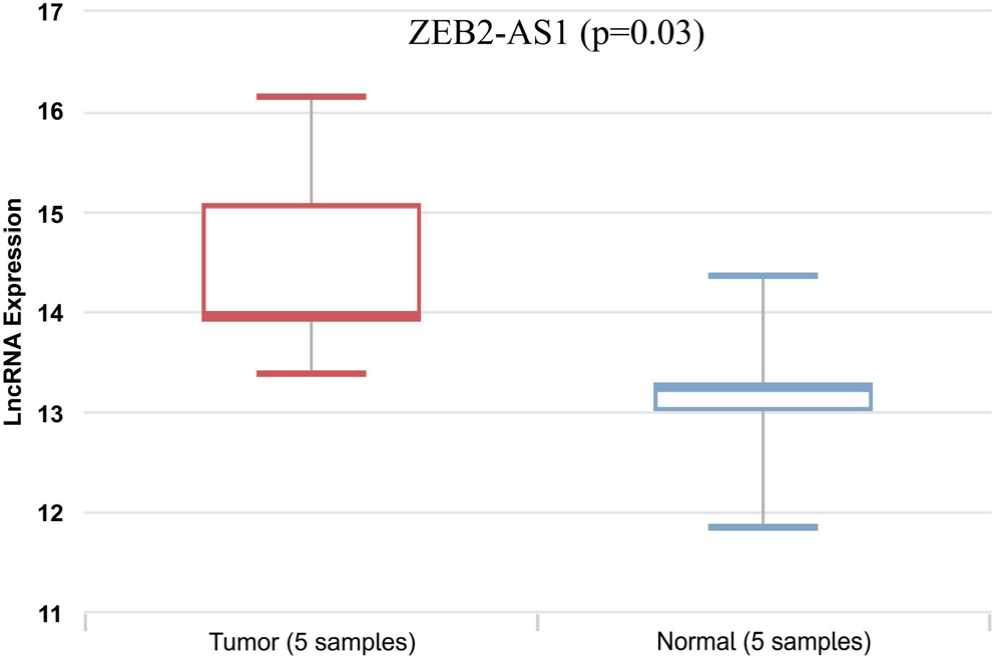
The expression profile of the ZEB2‐AS1 in breast cancer. The expression of ZEB2‐AS1 was determined in tumor versus normal tissues of BC by the lnCAR database. *p* < .05 was considered statistically significant.

**FIGURE 2:**
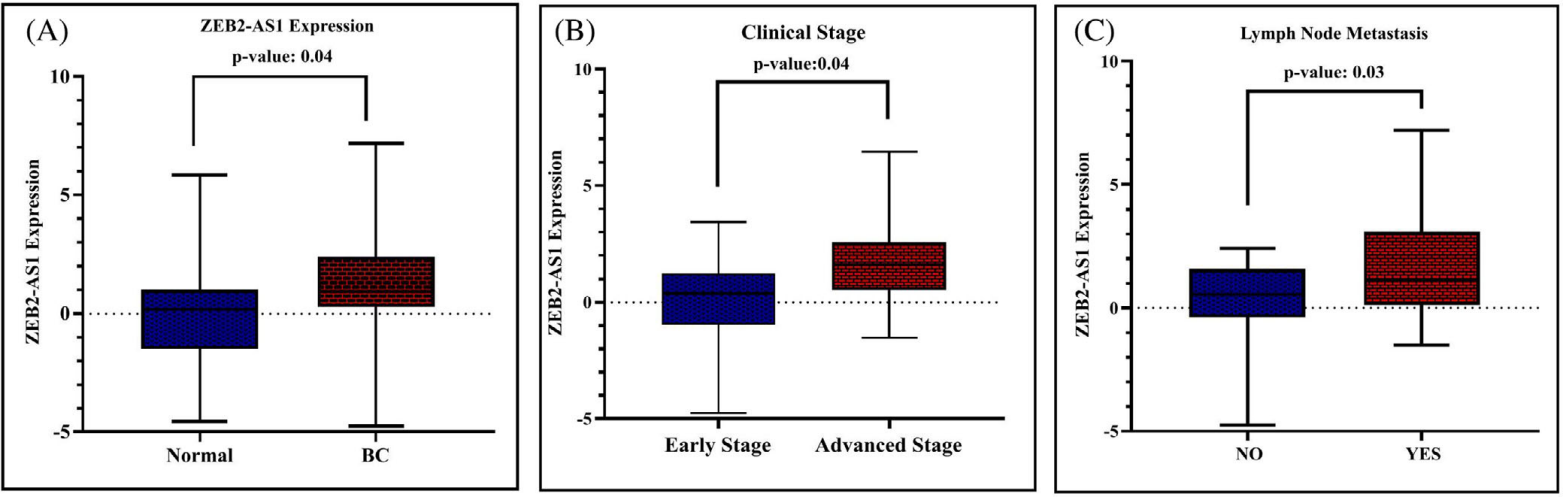
The relative expression of the ZEB2‐AS1 in tumor compared with matched normal tissues (A). The ZEB2‐AS1 mRNA level between cancer tissues with early‐stage and advanced‐stage samples (B) and differentially expressed lncRNA‐ZEB2‐AS1 in breast cancer specimens with and without lymph node metastasis (C). *p* < .05 was considered statistically significant.

A previous study has reported that ZEB2‐AS1 correlated positively with the progression of many tumors.^16^ To assess the ZEB2‐AS1 potential role in BC development, the LnCAR database was used. The expression analysis using LnCAR based on GEO study GPL15314 (platform)_GSE80266 (Series) demonstrated that the ZEB2‐AS1 transcriptional levels are elevated in 5 cancerous samples in comparison to 5 healthy tissues (*p* < .031), with the fold change of 1.34 (Figure 1). This database did not provide differential expression of ZEB2‐AS1 expression between the breast cancer stages though. Hence, we performed the baseline qRT‐PCR and detected expression in 30 pairs of BC tissues as well as the adjacent normal tissues for further confirmation. The result demonstrated that ZEB2‐AS1 expression is upregulated in BC tissues in comparison to adjacent normal tissues (*p* = .04; Figure 2A); according to our findings based on the LnCAR dataset. Furthermore, the correlation of ZEB2‐AS1 expression level was analyzed with patients' clinicopathological variables (Table 3). Overexpression of ZEB2‐AS1 was notably related to advanced stages of breast cancer (*p* = .04; Figure 2B) and lymph node metastasis (*p* = .03; Figure 2C). We could not find a correlation between the expression of ZEB2‐AS1 with the rest of the clinicopathological features.

**TABLE 3:** Result of association between genes expression and patient's features.

	ZEB2‐AS1		ZEB2		Vimentin		E‐cadherin	
	*n* (%)	*p*‐value	*n* (%)	*p*‐value	*n* (%)	*p*‐value	*n* (%)	*p*‐value
Age								
>50	14 (46.6)	.4	14 (46.6)	.7	14 (46.6)	.7	14 (46.6)	.3
<50	16 (53.3)		16 (53.3)		16 (53.3)		16 (53.3)	
Tumor size								
>5	8 (26.6)	.8	8 (26.6)	.6	8 (26.6)	.7	8 (26.6)	.2
<5	22 (73.3)		22 (73.3)		22 (73.3)		22 (73.3)	
Grade								
I & II	21 (70)	.3	21 (70)	.9	21 (70)	.5	21 (70)	.9
III & IV	9 (30)		9 (30)		9 (30)		9 (30)	
Stage								
I & II	12 (40)	**.04**	12 (40)	.1	12 (40)	.07	12 (40)	**.02**
III & IV	18 (60)		18 (60)		18 (60)		18 (60)	
ER								
Positive	24 (80)	.5	24 (80)	.9	24 (80)	**.01**	24 (80)	.2
Negative	6 (20)		6 (20)		6 (20)		6 (20)	
PR								
Positive	21 (70)	.6	21 (70)	.9	21 (70)	.3	21 (70)	.4
Negative	9 (30)		9 (30)		9 (30)		9 (30)	
HER								
Positive	18 (62)	.2	18 (62)	.5	18 (62)	.8	18 (62)	.9
Negative	11 (38)		11 (38)		11 (38)		11 (38)	
Lymph node metastasis								
Yes	16 (53.3)	**.03**	16 (53.3)	.4	16 (53.3)	.6	16 (53.3)	.6
No	14 (46.6)		14 (46.6)		14 (46.6)		14 (46.6)	
Necrose								.5
Yes	14 (46.6)	.2	14 (46.6)	.2	14 (46.6)	**.03**	14 (46.6)	
No	16 (53.3)		16 (53.3)		16 (53.3)		16 (53.3)	

Abbreviation: *n*, number.

### 3.2 In silico analysis of overall survival

**FIGURE 3:**
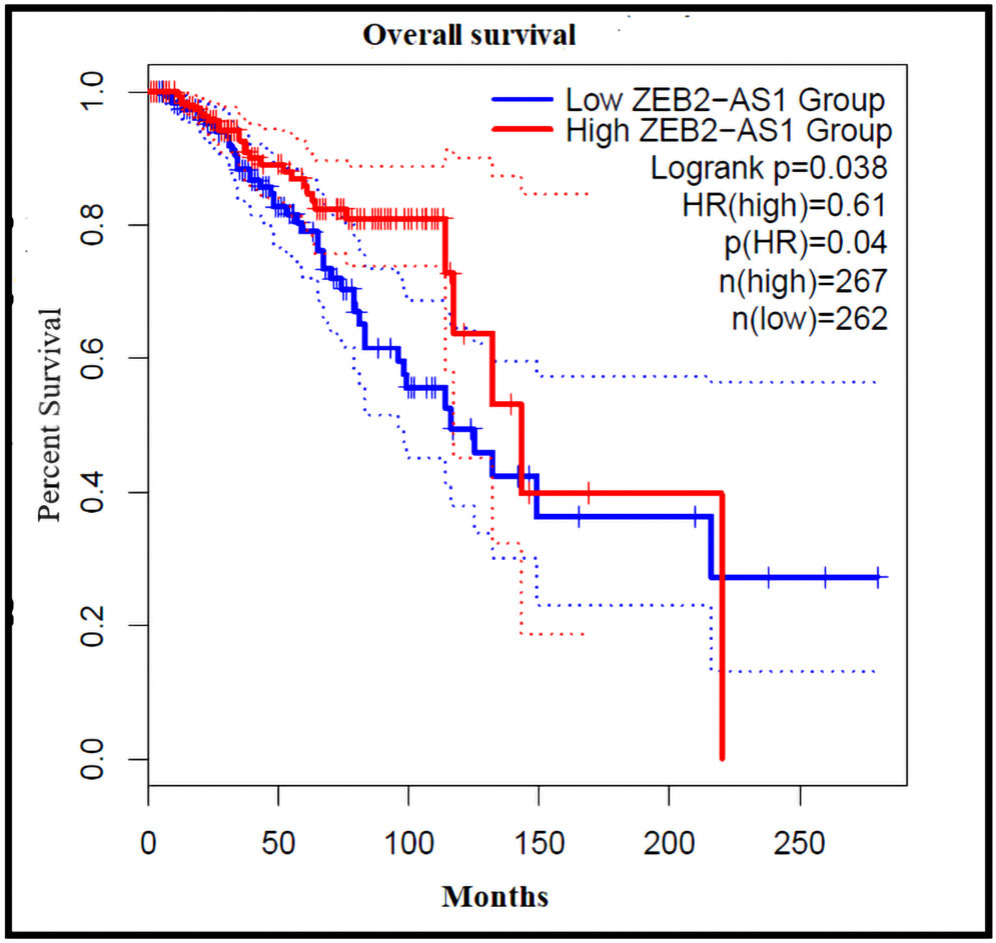
Survival curve evaluating the prognostic value of ZEB2‐AS1. Overall Survival curves have been plotted for patients with BC using the GEPIA2. A log‐rank *p* < .05 was considered statistically significant.

**FIGURE 4:**
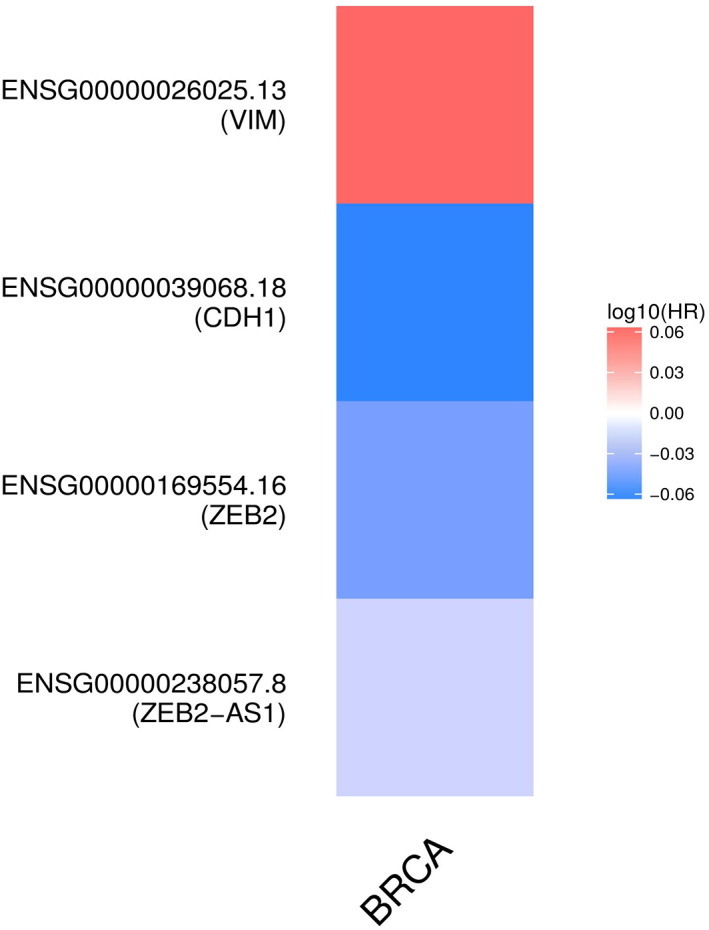
Survival analysis of ZEB2‐AS1, ZEB2, vimentin, and E‐cadherin in BC via gene expression analysis and survival duration based on TCGA data. High‐expression (75%) and low‐expression (25%) were considered as the expression threshold for splitting two (high vs. low) patient groups with BC. The highlighted box around the tiles displays statistical significance (*p* < .05). Red and blue colors had a poor and good prognosis, respectively, and the strength of the color identified the HR value.

We conducted a survival analysis using the ZEB2‐AS1 expression pattern and plotted the Kaplan–Meier curve. Evaluation of GEPIA2 results from BC patients demonstrated that ZEB2‐AS1 overexpression in BC tissues was remarkably related to a poor OS than ZEB2‐AS1 low expression (HR = 0.61, *p* = .038; Figure 3). Although there was a slight negative correlation between ZEB2‐AS1 expression and RFS, the analysis did not confirm it to be statistically significant (*p* = .25, HR = 0.74). In addition, we implemented the survival map module that compares the survival contribution of ZEB2‐AS1, ZEB2, vimentin, and E‐cadherin in BC. The OS results based on the cut‐off quartile group showed that ZEB2‐AS1 has the potential for prognostic value in breast cancer (Figure 4).

### 3.3 In silico analysis of correlation of ZEB2‐AS1 with ZEB2 and EMT markers

**FIGURE 5:**
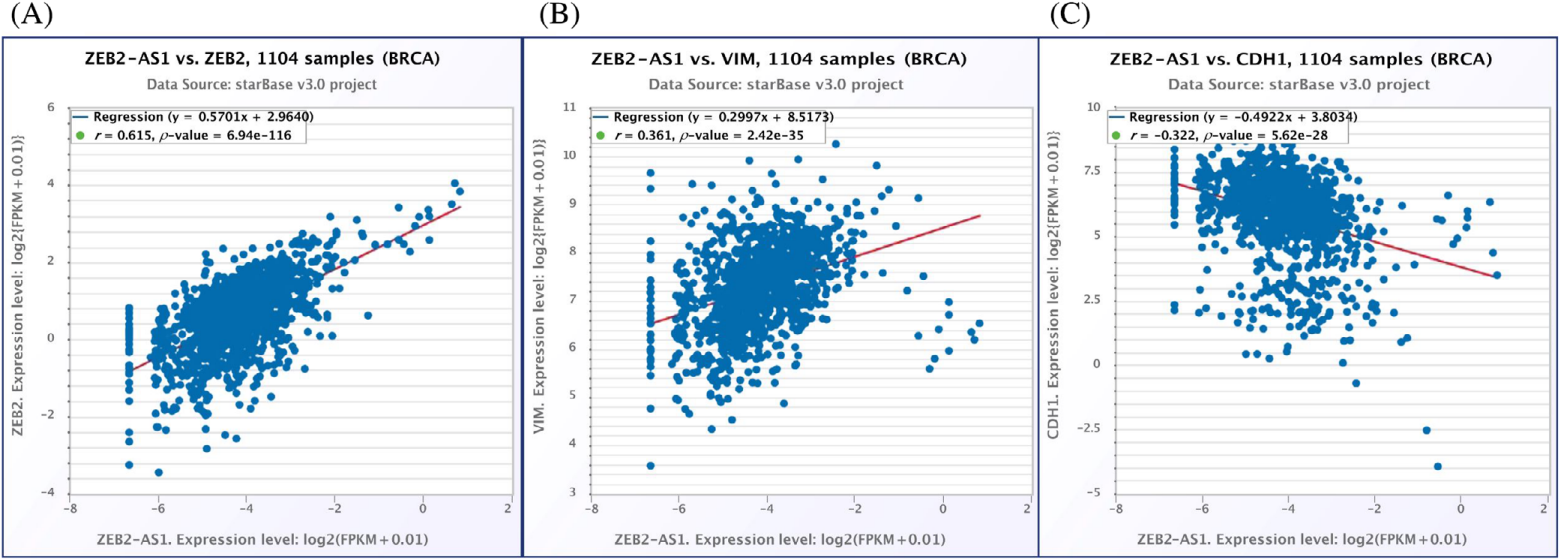
In silico analysis of the correlation between ZEB2‐AS1 and ZEB2 (A), vimentin (B), and E‐cadherin (C). *p* < .05 was considered statistically significant.

We assessed the correlation of ZEB2‐AS1 expression levels and ZEB2 and EMT markers in BC tissues using StarBase 3.0 online tool. Our data showed that ZEB2‐AS1 expression was correlated positively with ZEB2 (*r* = .61, *p* = 6.94e−116) and vimentin (*r* = .36, *p* = 2.42e−35), while negatively correlated with E‐cadherin (*r* = −.32, *p* = 5.62e−28) (Figure 5A–C).

### 3.4 Expression level of ZEB2 and EMT‐induce markers in BC tissues

**FIGURE 6:**
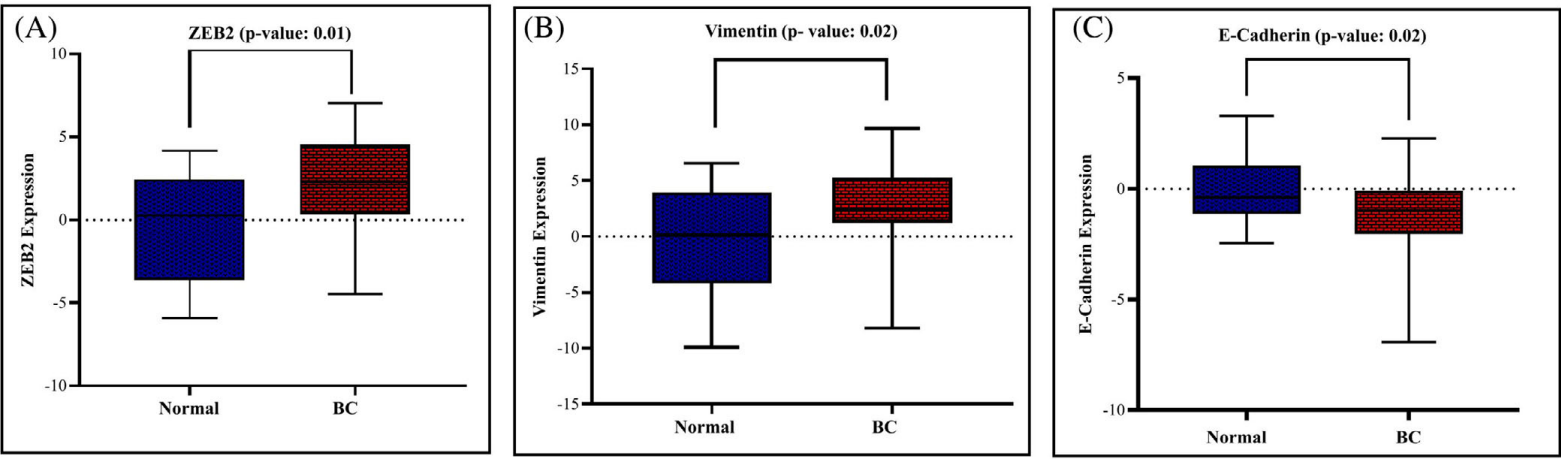
The expression level of E‐cadherin and EMT markers in BC tissues. The expression level of ZEB2 (A) and differentially expressed vimentin in breast cancer specimens (B) and E‐cadherin expression in breast cancer and normal tissues. *p* < .05 was considered statistically significant.

**FIGURE 7:**
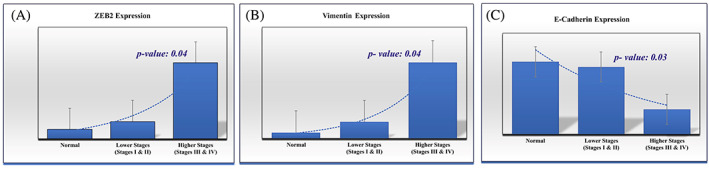
The EMT‐related markers mRNA level between cancer tissues with early‐stage and advanced‐stage samples. ZEB2 (A), vimentin (B), E‐cadherin (C). *p* < .05 was considered statistically significant.

According to previous investigations, we found that ZEB2‐AS1 is an antisense lncRNA that is transcribed antisense from the ZEB2 gene.^17^ ZEB2 acts as a transcription factor that can modulate E‐cadherin and vimentin.^18^, ^19^ ZEB2 protein has an inhibitory effect on the E‐cadherin gene which is a cell–cell junction protein. In addition, ZEB2 increases the expression of a mesenchymal factor called vimentin.^20^ In this research, we measured ZEB2, vimentin, and E‐cadherin expression levels via Quantitative RT‐PCR. These results revealed overexpression of mesenchymal factors ZEB2 (*p* = .01) and vimentin (*p* = .02) in breast cancer tissues in comparison to adjacent normal tissues. Furthermore, downregulation of E‐cadherin was observed in breast cancer tissues (*p* = .02) (Figure 6A–C). We also compared the expression of these genes between early‐stage and advanced‐stage breast cancer. We observed that the expression level of ZEB2 and vimentin were greater in advanced stage (III & IV) tissues (*n* = 18) compared with early stage (I & II) tissues (*p* = .04), (*p* = .04), but E‐cadherin expression was decreased in the higher stage of breast cancer (*p* = .03) (Figure 7A–C).

### 3.5 Correlation of ZEB2‐AS1 with ZEB2 and EMT markers

**FIGURE 8:**
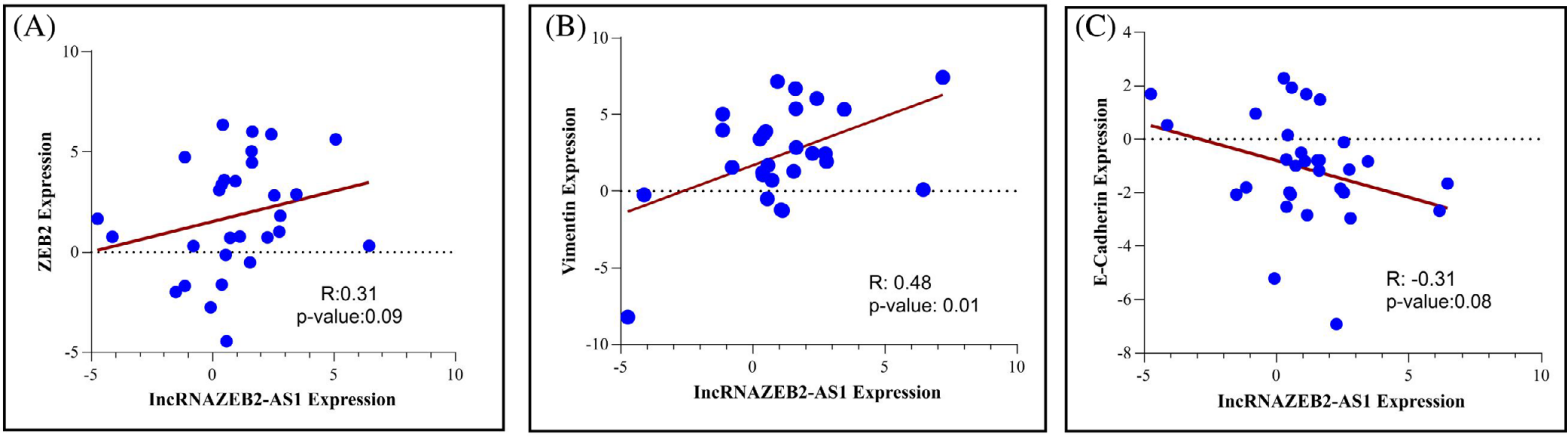
Clinical relevance of lncRNA‐ZEB2‐AS1 between ZEB2 and EMT‐related markers in human breast cancer. Correlation between lncRNA‐ZEB2‐AS1 and ZEB2 (A) and correlation between lncRNA‐ZEB2‐AS1 and vimentin (B) and correlation between lncRNA‐ZEB2‐AS1 and E‐cadherin (C). *p* < .05 was considered statistically significant.

We evaluated the clinical correlation of ZEB2‐AS1 transcriptional levels with ZEB2 and EMT markers in BC tissues using the qRT‐PCR technique. The result indicated that ZEB2‐AS1 was positively correlated with ZEB2 and vimentin, while a negative association was observed between ZEB2‐AS1 and E‐cadherin (Figure 8). Taken together, our findings revealed that ZEB2‐AS1 expression level was associated with vimentin, but this correlation was not significant with ZEB2 and E‐cadherin in BC patients (Table 4).

**TABLE 4:** Correlation between ZEB2‐AS1 with EMT‐related marker.

Correlation between …		*R* value (95% confidence interval)	*p*‐value
LncRNA ZEB2‐AS1	ZEB2	0.31 (−0.06870 to 0.6186)	.09
LncRNA ZEB2‐AS1	E‐cadherin	−0.31 (−0.6015 to 0.04523)	.08
LncRNA ZEB2‐AS1	Vimentin	0.48 (0.1244 to 0.7284)	.01

### 3.6 ROC curve analysis

**FIGURE 9:**
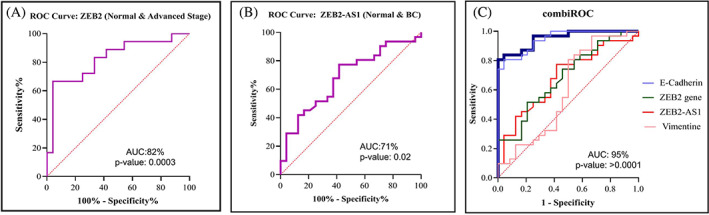
The diagnostic power of ZEB2 and ZEB2‐AS1 expression in BC by using ROC curve analysis. ROC curve analysis with respect to the ZEB2 gene expression in distinguishing between the early stage and the final stage of breast cancer (A). ROC curve analysis with respect to lncRNA‐ZEB2‐AS1 was implemented to discriminate between tumor and normal tissues of breast cancer (B). Combined evaluating the diagnostic power of ZEB2‐AS1, ZEB2, vimentin, and E‐cadherin gene expression in determining tumor and normal tissues in breast cancer employing CombiROC analysis. A considerable enhancement in AUC (95%), as well as enhanced sensitivity and specificity, was observed. *p* < .05 was considered statistically significant (C).

We plotted the ROC curve using the true positive fraction (sensitivity) versus false‐positive fraction (1‐specificity) across different cutoff points. The diagnostic capacity of genes was evaluated by computation of the area under the curve (AUC) estimates. Among the four genes under study, ZEB2 showed the best diagnostic potential to discriminate between the early stage and end‐stage (AUC value: 82%) (Figure 9A). The AUC value for lncRNA ZEB2‐AS1 was 71% (Figure 9B). Moreover, the combination of ZEB2‐AS1, ZEB2, vimentin, and E‐Cadherin increased the AUC to 95%. The results of the ROC curve analysis are shown in Figure 9C.

## 4 DISCUSSION

LncRNA ZEB2‐AS1 is a recently found lncRNA. Therefore, we have a poor contemplation over the biological function of this lncRNA in BC. The increased expression and the oncogenic function of ZEB2‐AS1 were confirmed in several cancers, including hepatocellular carcinoma,^21^ bladder cancer,^9^ pancreatic cancer,^22^ colorectal cancer,^23^ and triple‐negative breast cancer.^11^ Studies on the role of ZEB2‐AS1 in cancer progression are scarce and this concept has remained quite unclear so far. There are not bioinformatics analyses about ZEB2‐AS1 expression and its prognostic value of that. In the present research, the bioinformatics analysis was run to quickly assess ZEB2‐AS1 expression in breast cancer at the mRNA level. Accordingly, lnCAR datasets show ZEB2‐AS1 overexpression in breast cancer tissues in comparison to normal tissues (*p* = .03). Furthermore, by examining the expression of ZEB2‐AS1 in breast cancer tumor tissues and comparing it with normal tissues by RT‐PCR technique, it was confirmed that ZEB2‐AS1 is upregulated in tumor tissues (*p* = .04).

Moreover, the elevated expression was associated with a higher stage and lymph node metastasis (lnCAR database does not provide information on ZEB2‐AS1 differential expression between BC stages). One study in triple‐negative breast cancer determined increased ZEB2‐AS1 expression in patients with lymph node metastasis compared with patients without lymph node metastasis.^11^ This study accordance with our results but we performed the present study on different samples that were ER‐positive or PR‐ positive.

In order to examine the survival analysis, we used the GEPIA2 database. Our finding supported that ZEB2‐AS1 overexpression is associated with poor OS and RFS, suggesting an oncogenic function of ZEB2‐AS1 in breast cancer pathogenesis. It is also highlighted that ZEB2‐AS1 overexpression has an association with a higher risk of tumor metastasis and tumor progression. Previous studies indicated that high ZEB2‐AS1 expression was associated with shorter overall survival in HCC,^21^ and AML patients.^17^

ZEB2 gene can be regulated by several non‐coding RNAs in cancers. For example, Gennaro et al.^24^ reported that P53 mRNA can restrict ZEB2 expression by inducing the transcription of miR30a. In addition, Li et al.^25^ speculated that OIP5‐AS1 boosts breast cancer metastasis by upregulating ZEB2 mRNA.

Vimentin is a mesenchymal marker that plays roles in various cancers and is targeted by different lncRNAs in cancers and facilitates EMT. Liao et al.^26^ revealed that lncRNA H19 increases metastasis and EMT via the upregulation of vimentin in lung cancer. Qian et al.^27^ investigated this in colorectal cancer samples and found that LINC00857 upregulates vimentin by sponge miR‐1306 and promotes EMT.

Past studies have shown that EMT is the procedure in that epithelial cells lose their polarity and cellular connections and acquire mesenchymal properties.^28^ In addition, dysregulation in EMT‐markers expression occurs in the cell, leading to EMT and tumor metastasis. The TGF‐β pathway is a cellular pathway involved in the development of EMT in cancers. Activation of the TGF‐*β* pathway increases the EMT‐marker transcription such as Snail1 and ZEB2.^29^ Snail is a transcription factor that stimulates the transcription of lncRNA ZEB2‐AS1.^30^ LncRNA ZEB2‐AS1 is a lncRNA that is transcribed towards antisense from ZEB2 and can regulate the ZEB2 gene via binding to ZEB2 mRNA and prevent the removal of an intron in 5′‐UTR. The present ZEB2 intron is actually an internal ribosome entry site (IRES) to increase the translation of this gene. As a pivotal transcription factor, ZEB2 may serve a tumor progression function in cancers, especially in EMT.^12^ Increased ZEB2 expression inhibits transcription of E‐Cadherin and promotes vimentin expression, which is a mesenchymal factor.

The current research evaluated the transcriptional level of EMT markers in BC, and it revealed that ZEB2 and vimentin gene expression levels in tumor samples had significant increases compared to normal samples. E‐cadherin expression showed a significant decrease in tumor tissues. In addition, the difference in the expression level of EMT markers was investigated between breast cancer stages, and we found that ZEB2 and vimentin expression was highly upregulated in the end‐stage BC patients compared with early‐stage of cancer. Moreover, the epithelial factor E‐Cadherin expression in BC tissues of higher tumor stages was less than in the lower‐grade tissues. These data suggested that overexpression of mesenchymal factors and downregulation of epithelial factors may induce EMT in breast cancer, and ZEB2‐AS1 might be contributing to this process by regulating the EMT‐related transcripts. Wang et al.^10^ performed a study on gastric cancer cells and they found that knockdown of ZEB2‐AS1 decreased Wnt/*β*‐Catenin pathway activity via reducing the ZEB2 gene. Their results suggested that ZEB2‐AS1 up‐regulation activated the Wnt/*β*‐Catenin pathway through augmented ZEB2 expression in gastric cancer cells. That is in accordance with our results on breast cancer.

Previous studies have explored the correlation of ZEB2‐AS1 with EMT‐related markers in head and neck squamous cell carcinoma (HNSCC) and triple‐negative BC and showed a positive association between ZEB2‐AS1 and ZEB2/vimentin. In addition, these studies illustrated that ZEB2‐AS1 has a negative correlation with E‐cadherin.^11^, ^31^ In the present research, a correlation study of ZEB2‐AS1 and EMT‐related markers indicated that ZEB2‐AS1 has a positive correlation with ZEB2 and vimentin, whereas there is a negative correlation between ZEB2‐AS1 and E‐cadherin, but this association was not statically significant (ZEB2; E‐cadherin), which might be due to the insufficient sample size. The ROC curve analysis suggested a diagnostic potential for the ZEB2 gene in discriminating the stages of cancer and probably metastasis in breast cancer. In addition, combination ROC indicated that these genes (ZEB2‐AS1, ZEB2, vimentin, and E‐cadherin) could have the potential to diagnostic of breast cancer metastasis. However, more research is needed to confirm this finding. We had limitations in collecting samples, and due to ZEB2‐AS1 being a recently discovered lncRNA, there was little information about this in a variety of databases.

## 5 CONCLUSION

In conclusion, our findings showed that lncRNA ZEB2‐AS1 up‐regulation could act on breast cancer onset and development through ZEB2, vimentin, and E‐cadherin dysregulation. This lncRNA probably has a diagnostic and prognostic characterization in breast cancer metastasis and it seems to be a good therapeutic and pharmaceutics value to prevent breast cancer progression. Therefore, these four genes may be used in putative diagnostic panels to differentiate the malignant condition and metastasis of breast tissues. Although, more functional studies are required to validate our findings.

## AUTHOR CONTRIBUTIONS

**Mahboobeh Zarei:** Conceptualization (lead); methodology (lead); resources (lead); writing – original draft (lead). **Kianoosh Malekzadeh:** Data curation (equal); investigation (equal); visualization (equal). **Mahmoud Omidi:** Formal analysis (equal); investigation (equal); visualization (equal). **Pegah Mousavi:** Funding acquisition (lead); project administration (equal); supervision (lead); writing – review and editing (lead).

## FUNDING INFORMATION

This study received a grant (#980059) from the deputy of Research in the Hormozgan University of Medical Sciences, Bandar Abbas, Iran.

## CONFLICT OF INTEREST STATEMENT

The authors have stated explicitly that there are no conflicts of interest in connection with this article.

## ETHICS STATEMENT

This research was approved by the ethics committee of Hormozgan University of Medical Science (Ethical code: #IR.HUMS.REC.1398.038).

## Data Availability

The data that support the findings of this study are available from the corresponding author upon reasonable request.
